# Integrative Proteomic Analysis of Multiple Posttranslational Modifications in Inflammatory Response

**DOI:** 10.1016/j.gpb.2020.11.004

**Published:** 2021-03-02

**Authors:** Feiyang Ji, Menghao Zhou, Huihui Zhu, Zhengyi Jiang, Qirui Li, Xiaoxi Ouyang, Yiming Lv, Sainan Zhang, Tian Wu, Lanjuan Li

**Affiliations:** 1 State Key Laboratory for Diagnosis and Treatment of Infectious Diseases, National Clinical Research Center for Infectious Diseases, Collaborative Innovation Center for Diagnosis and Treatment of Infectious Diseases, The First Affiliated Hospital, College of Medicine, Zhejiang University, Hangzhou 310003, China; 2 The First Affiliated Hospital, College of Medicine, Zhejiang University, Hangzhou 310003, China; 3 Department of Colorectal Surgery, Sir Run Run Shaw Hospital, College of Medicine, Zhejiang University, Hangzhou 310003, China; 4 Quzhou Second People’s Hospital, Quzhou 324000, China

**Keywords:** Proteome, Crosstalk, Inflammation, Lipopolysaccharide, Macrophage

## Abstract

Posttranslational modifications (PTMs) of proteins, particularly acetylation, phosphorylation, and ubiquitination, play critical roles in the host innate immune response. PTMs’ dynamic changes and the **crosstalk** among them are complicated. To build a comprehensive dynamic network of **inflammation**-related proteins, we integrated data from the whole-cell **proteome** (WCP), acetylome, phosphoproteome, and ubiquitinome of human and mouse **macrophages**. Our datasets of acetylation, phosphorylation, and ubiquitination sites helped identify PTM crosstalk within and across proteins involved in the inflammatory response. Stimulation of macrophages by **lipopolysaccharide** (LPS) resulted in both degradative and non-degradative ubiquitination. Moreover, this study contributes to the interpretation of the roles of known inflammatory molecules and the discovery of novel inflammatory proteins.

## Introduction

Macrophages are resident phagocytic cells which act as effector cells in the innate immune system and play a crucial role in initiating adaptive immunity by recruiting other immune cells [1]. Located throughout the body, macrophages are among the first defensive cells to interact with foreign or abnormal host cells and their products. They are the most efficient phagocytes that can ingest and process foreign materials, dead cells, and debris; they also release various secretory products to mobilize other host cells and influence other resident cells in the inflammatory response [2].

Posttranslational modifications (PTMs) have been reported to play a critical role in regulating multiple inflammatory signaling pathways. Phosphorylation, polyubiquitination, and acetylation exert diverse effects on pathogen recognition receptor-dependent inflammatory responses [3]. Phosphorylation is a widely investigated type of PTMs in innate immunity [4]. It is catalyzed by protein kinases and reversed by protein phosphatases. The phosphorylation and dephosphorylation of certain proteins regulate the activation and inactivation of many Toll-like receptor (TLR)-dependent signaling molecules, such as mitogen activated protein kinases (MAPKs), inhibitor of nuclear factor kappa B alpha (IκBα), IκB kinase alpha (IKKα), IκB kinase beta (IKKβ), and interferon regulatory factor 3 (IRF3) [5]. Existing research also recognizes the critical role of the phosphorylation of certain innate immune adaptor proteins. For example, the phosphorylation of mitochondrial antiviral signaling protein (MAVS), stimulator of interferon genes (STING), and Toll/IL-1R domain-containing adaptor inducing interferon-beta (TRIF) is necessary for recruiting IRF3 to activate type I IFN production, which is essential for the activation of antiviral immunity [6]. Protein ubiquitination also plays a pivotal role in several immunological processes [7]. Ubiquitin, a highly conserved polypeptide comprising 76 amino acids, is attached to substrates by a three-step enzymatic cascade [8]. Seven lysine residues within ubiquitin can be bonded with polyubiquitin chains, namely, K6, K11, K27, K29, K33, K48, and K63. Ubiquitin can also be ubiquitinated at the N-terminal methionine residue (M1) [3], [9]. Different types of ubiquitin connections lead to different outcomes. For example, K48-linked polyubiquitination of IκBα results in its proteasomal degradation, promoting the nuclear translocation of NF-κB [10]. In contrast, K63-linked polyubiquitination of TAK1-binding protein 2/3 (TAB2/3), tumor necrosis factor (TNF) receptor-associated factor 6 (TRAF6), NF-κB essential modulator (NEMO), and TNF receptor-associated factor 3 (TRAF3) is proteasome-independent but a requisite for activating NF-κB and IRF3 [3]. Furthermore, M1-, K11-, K48-, and K63-linked ubiquitin chains are not only involved in the TNF-induced inflammatory signaling pathway, but also play critical roles in the regulation of the downstream signaling cascade [8]. In addition to these two widespread PTMs, several studies have highlighted the important roles of other PTMs, including acetylation, in regulating immune responses. The change of chromatin structure via the acetylation of histones and its influence on gene transcription are well understood [11], [12]. For example, in antiviral immune response, histone deacetylase 9 (HDAC9) is up-regulated and in turn activates TANK-binding kinase 1 (TBK1) by deacetylation, leading to increased IFN production [13]. Furthermore, acetylation has been discovered in many non-histone substrates which participate in immune response [14].

In the past few years, evidence for comprehensive crosstalk between PTMs has accumulated. An example of this crosstalk is the NF-κB signaling pathway, where IKKβ phosphorylates IκBα, resulting in the K48-linked polyubiquitination and degradation of IκBα, the subsequent release of NF-κB, and the entry of p65 and p50 dimers into the nucleus to activate target genes. Also, some E3 ubiquitin ligases must be phosphorylated to become catalytically active or can only ubiquitinate the phosphorylated forms of their substrates [15]. According to a recent study by Huai et al. [16], phosphorylation, ubiquitination, and acetylation occur in the same protein. In more detail, IRF3 activity is strongly regulated by PTMs, such as ubiquitination and phosphorylation; however, lysine acetyltransferase 8 (KAT8) inhibits antiviral immunity by acetylating IRF3 [16]. The combination of various PTMs in one protein apparently creates a “PTM code” that is recognized by specific effectors to initiate or inhibit downstream events [17].

Although various studies have investigated the role of PTMs in innate immunity, few quantitative analyses of PTMs have been conducted in this area. Furthermore, the crosstalk between different PTMs is always a difficult subject to explore, as PTMs usually occur too rapid to be measured. Here, we selected two macrophage cell lines, RAW264.7 macrophages from mice and THP-1 macrophages from human, to establish a model of the inflammatory response induced by lipopolysaccharide (LPS) stimulation. We applied high-resolution mass spectrometry (MS) to discover and quantify the changes in phosphorylation, ubiquitination, and acetylation during the innate immune responses at 0.5 h and 2 h after LPS stimulation. Using the advanced stable isotope labeling with amino acids in cell culture (SILAC) technique [18] and the unsupervised clustering method, we successfully profiled all three PTMs with high accuracy and divided PTM events into different groups for subsequent analyses. Here, we present one of the first investigations into PTM crosstalk during the innate immune response. Our findings could make an irreplaceable contribution to the field studying novel changes in PTMs occurring during inflammation.

## Results

### Workflow of the integrative proteomic analysis of LPS-stimulated macrophages

**Figure 1:**
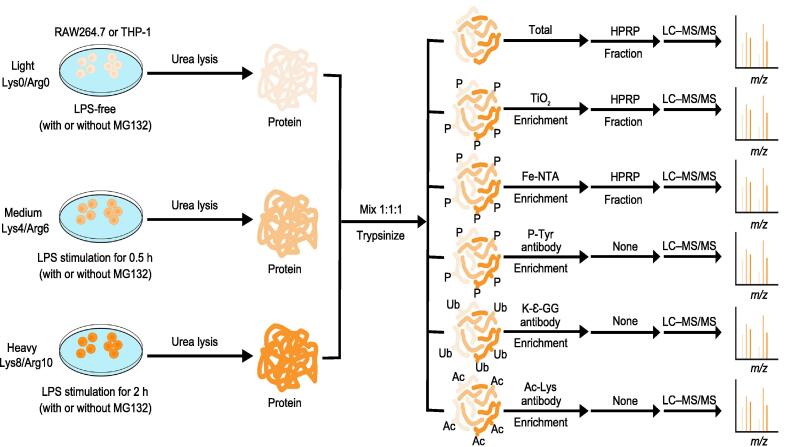
**Workflow of the integrative proteomic analysis of macrophages stimulated with LPS** RAW264.7 cells from mice or THP-1 cells from human cultivated in light, medium, or heavy SILAC media were stimulated with LPS at different time points (0.5 h or 2 h) with or without MG132 treatment. THP-1 cells were treated with 20 ng/ml PMA for 16 h before LPS stimulation. The ‘rested’ and ‘stimulated’ cells were then prepared for whole-cell proteome, phosphoproteome, ubiquitinome, and acetylome analyses. Different colors of cells, proteins, and peptides represent light, medium, and heavy SILAC labels. SILAC, stable isotope labeling with amino acids in cell culture; LPS, lipopolysaccharide; PMA, phorbol 12-myristate 13-acetate; P, phosphoproteome; Ac, acetylome; Ub, ubiquitinome; HPRP, high pH reversed-phase chromatography; LC–MS/MS, liquid chromatography–tandem mass spectrometry.

To investigate the crosstalk of the PTMs of various proteins involved in inflammation, we examined the changes in the acetylome, phosphoproteome, and ubiquitinome in two inflammatory cell lines (RAW264.7 from mice and THP-1 from human) at 0.5 h and 2 h after LPS stimulation. Quantitative profiling of acetylation sites was performed using Ac-Lys proteomics. Ac-Lys peptides generated by tryptic cleavage of an acetylated lysine substrate can be enriched with the Ac-Lys antibody. In addition, the changes in the phosphoproteome were quantified using the TiO_2_, Fe-NTA, and P-Tyr methods: TiO_2_ and Fe-NTA are complementary methods to enrich phosphorylation sites in serine, threonine, and tyrosine substrates, while the P-Tyr antibody specifically enriches phosphorylation sites in tyrosine substrates. We also performed quantitative profiling of ubiquitination sites using K-Ɛ-GG proteomics. K-Ɛ-GG remnants generated by tryptic cleavage of an ubiquitinated lysine substrate can be enriched with the K-Ɛ-GG antibody. We applied SILAC for the relative quantification of PTM sites, and all experiments were performed in three biological replicates. In addition, we used high pH reversed-phase chromatography (HPRP) to increase the number of identified proteins and PTM sites (Figure 1). After comparing the three phosphorylation enrichment methods, approximately one-third to half of the phosphorylation sites were quantified by both TiO_2_ and Fe-NTA methods. On the other hand, most of the phosphorylation sites quantified using P-Tyr method were different from those identified by the other two methods (Figure S1A). The effects of the proteasome inhibitor MG132 on the identification of proteins and ubiquitination sites were assessed. We found that MG132 had little effect on the identification of proteins in the whole-cell proteome (WCP), but exerted a relatively significant effect on the identification of ubiquitination sites (Figure S1B).

### The integrative proteomic analysis of LPS-stimulated macrophages showed high-confidence

**Figure 2:**
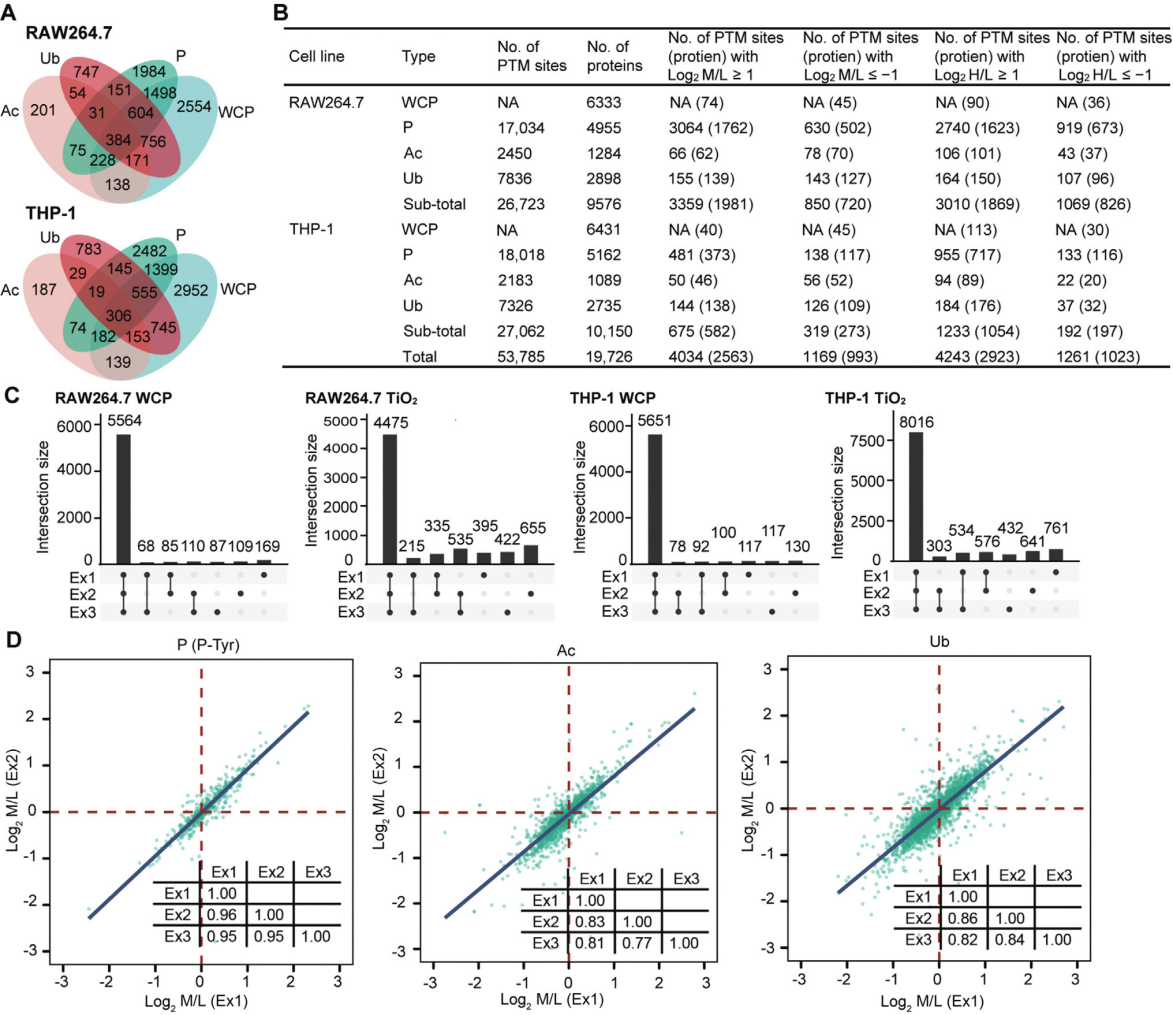
**The integrative proteomics data from LPS-stimulated macrophages had high-confidence** **A.** Venn diagrams of proteins quantified in WCP and in three types of PTM omics. **B.** Number of PTM sites and corresponding proteins quantified in PTM omics and WCP from RAW264.7 and THP-1 cells. **C.** Number of proteins and phosphorylation sites quantified in 1–3 biological replicates of WCP and TiO_2_ experiments, respectively, in RAW264.7 and THP-1 cells. **D.** Pearson’s correlation plots for two representative experiments (Ex1 and Ex2) analyzing P-Tyr, Ac, and Ub in RAW264.7 cells. The inserted tables show Pearson’s correlation coefficients for all three biological replicates. WCP, whole-cell proteome; PTM, posttranslational modification; L, light SILAC label; M, medium SILAC label; H, heavy SILAC label; NA, not available.

To determine whether the target proteins of different PTMs differed, we analyzed the overlaps of proteins identified by three types of PTM proteomes and WCP. As shown in Figure 2A, approximately half of the proteins identified in WCP did not contain any PTM sites (2554 proteins in RAW264.7 cells and 2952 proteins in THP-1 cells). In contrast, 384 proteins in RAW264.7 cells and 306 proteins in THP-1 cells carried all three types of PTMs. Figure 2B provides an overview of the proteins and PTM sites quantified in our study. We quantified 6333 proteins in RAW264.7 cells and 6431 proteins in THP-1 cells, 2450 acetylation sites in 1284 proteins in RAW264.7 cells and 2183 acetylation sites in 1089 proteins in THP-1 cells, 17,034 phosphorylation sites in 4955 proteins in RAW264.7 cells and 18,018 phosphorylation sites in 5162 proteins in THP-1 cells, and 7836 ubiquitination sites in 2898 proteins in RAW264.7 cells and 7326 ubiquitination sites in 2735 proteins in THP-1 cells (Table S1). The upset plots shown in Figure 2C and Figure S1C showed the overlap between proteins quantified in three biological replicates in the WCP experiments and the overlap between PTM sites in three biological replicates in PTM proteomics experiments in a matrix layout, allowing us to easily validate the reproducibility of the experiments. Pearson’s correlation coefficients for pair-wise comparisons of the log_2_-transformed medium/light (M/L) abundances were up to 0.96 in RAW264.7 cells and 0.88 in THP-1 cells (Figure 2D, Figure S1D). These results indicate that our experiments are reproducible between biological samples.

### Different types of PTMs presented different properties and dynamic changes over time

**Figure 3:**
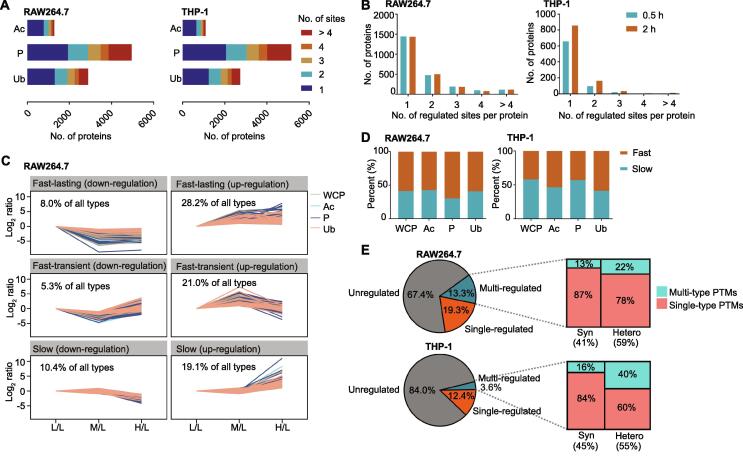
**Properties and dynamic changes among different types of PTM proteomes** **A.** Distribution of quantified PTM sites detected in identical proteins in the RAW264.7 and THP-1 cells. **B.** Distribution of all regulated PTM sites per protein in macrophages stimulated with LPS for 0.5 h or 2 h. **C.** Changes in integrative proteomics data in the LPS-treated RAW264.7 cells over time. Regulated proteins and PTM sites were clustered into the six categories using the fuzzy c-means method. “ratio” means the value of L/L, M/L, or H/L. **D.** Distribution of the regulated proteins in different PTM types. The regulated proteins were classified into slow and fast groups according to the speed of change in the PTM sites. **E.** Proteins were classified into unregulated, single-regulated, and multi-regulated groups according to the number of regulated PTM sites that each protein contained. The multi-regulated proteins were classified as “Syn” and “Hetero” depending on whether all the regulated PTM sites were in the same slow or fast group classified in (D). Syn, synchronous; Hetero, heterochronous.

The abundances of different types of PTMs on the same protein differed. As shown in Figure 3A, phosphorylated proteins usually contained more than one phosphorylation site (61% in RAW264.7 cells and 60% in THP-1 cells). The percentage of multiple PTM sites in phosphorylated proteins was higher than that in acetylated (39% in RAW264.7 cells and 40% in THP-1 cells) and ubiquitinated proteins (55% in RAW264.7 cells and 55% in THP-1 cells). We defined the PTM sites with an average 2-fold change (up- or down-regulation; *n* = 3) after 0.5 h or 2 h LPS stimulation as regulated PTM sites. Specifically, ∼ 1500 proteins in RAW264.7 cells and ∼ 700 proteins in THP-1 cells only contained one regulated PTM site, accounting for a larger proportion than those proteins containing more one regulated PTM site (Figure 3B). Notably, no proteins contained more than two regulated acetylation sites (Figure S2A).

We next analyzed the dynamic changes of PTM sites after 0.5 h or 2 h LPS stimulation. To this aim, we classified the regulated PTM sites into six clusters using the fuzzy c-means method. First, we divided the regulated PTM sites according to the speed at which changes occurred: the PTM sites that did not change after 0.5 h LPS stimulation were assigned to the slow-change group, while the PTM sites that changed after 0.5 h LPS stimulation were assigned to the fast-change group. Then, we divided the fast-change group into lasting and transient groups according to the persistence of the change after 2 h LPS stimulation. Finally, we spit each group into up-regulated and down-regulated groups, resulting in six clusters. The trends of changes in PTMs in the six clusters are displayed in Figure 3C (for RAW264.7 cells) and Figure S2B (for THP-1 cells). We found that the proportions of “slow” and “fast” items varied in different PTM types (Figure 3D, Figure S2C). We defined the proteins with at least a 2-fold change (up- or down-regulation) in the PTM sites during LPS stimulation as regulated proteins. The regulated proteins were then classified into single-regulated and multi-regulated groups according to the number of regulated PTM sites. In this study, the term “synchronous” was used to refer to the multi-regulated proteins in which the regulated PTM sites were in the same slow or fast group mentioned above. By contrast, the term “heterochronous” was used to describe the multi-regulated proteins in which the regulated PTMs occurred at different speeds. Figure 3E provides the summary statistics of the identified proteins classified using these standards. Taking the RAW264.7 cells as an example, a large proportion (67.4%) of the proteins detected did not contain any regulated PTM sites, while 19.3% of the proteins contained a single-regulated PTM site. Of the 13.3% of the proteins containing multi-regulated PTM sites, 41% were synchronous, and 59% were heterochronous. Moreover, 13% and 22% of the synchronous and heterochronous proteins contained multi-type PTMs.

### PTM crosstalk existed both within and across proteins

**Figure 4:**
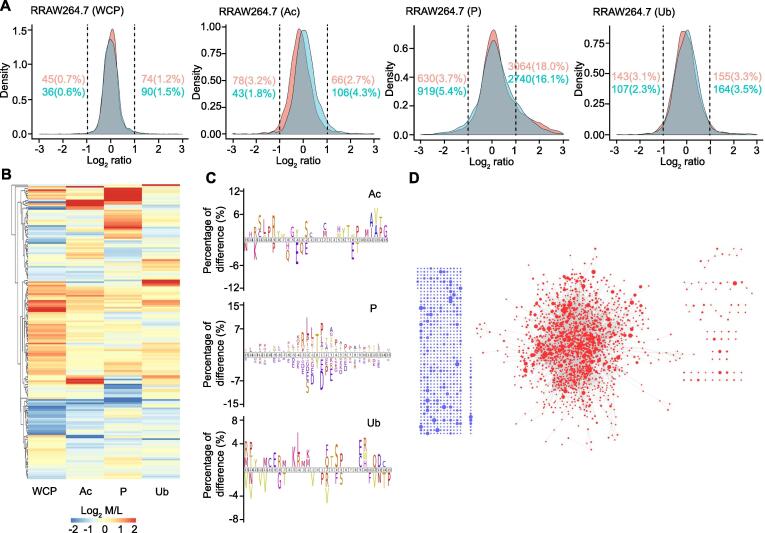
**Crosstalk between PTM sites on different proteins** **A.** Density gradient diagram of the Log_2_ ratio of proteins in WCP and the Log_2_ ratio of PTM sites in different PTM proteomes of RAW264.7 cells. Orange and cyan represent 0.5 h and 2 h after LPS stimulation, respectively. Orange and cyan numbers (percentages) on the left and right represent the number and percentage of regulated proteins/PTM sites at the two time points, respectively. **B.** Heatmap showing the Log_2_ M/L values of proteins quantified in both the WCP and all three PTM proteomes in RAW264.7 cells. Only proteins with Log_2_ M/L ≥ 1 or ≤ −1 are shown, and the colors of proteins in PTM proteomes indicate the mean Log_2_ M/L value of all PTM sites in the protein. **C.** iceLogo plots showing the difference of amino acid frequency at positions flanking the LPS-regulated PTM sites compared to unregulated PTM sites with *P* ≤ 0.05 in RAW264.7 cells. **D.** Interaction network for proteins with regulated PTM sites in RAW264.7 cells. Blue dots indicate the proteins with no interacting partner, while red dots indicate the proteins interacting with other proteins. The size of the dot indicates the number of regulated PTM sites.

We next investigated the overall distribution of changes in PTMs. As shown in Figure 4A and Figure S3A, more up-regulated PTM sites were identified than down-regulated sites in all types of PTMs in both cell lines. The heatmaps of regulated proteins quantified in both the WCP and all three PTM proteomes are also shown in Figure 4B and Figure S3B. We performed an iceLogo analysis to visualize the conserved patterns of different protein sequences between regulated and unregulated PTM sites (Figure 4C, Figure S3C). Using the STRING database, we also visualized the comprehensive protein–protein interactions between the regulated proteins (Figure 4D, Figure S3D; Table S2). PTM crosstalk across proteins was based on the assumption that if there is an interaction between two proteins, there is a higher probability that the PTM sites on these two proteins exist crosstalk, especially, when these PTM sites are regulated in a biological process. We found that phosphorylation and acetylation mainly targeted proteins related to chromatin assembly and mRNA processing, while ubiquitination mainly targeted proteins related to protein stability (Figure S4). Most of the identified proteins interacted with other proteins to some extent, indicating the complicated crosstalk among PTMs from different proteins.

**Figure 5:**
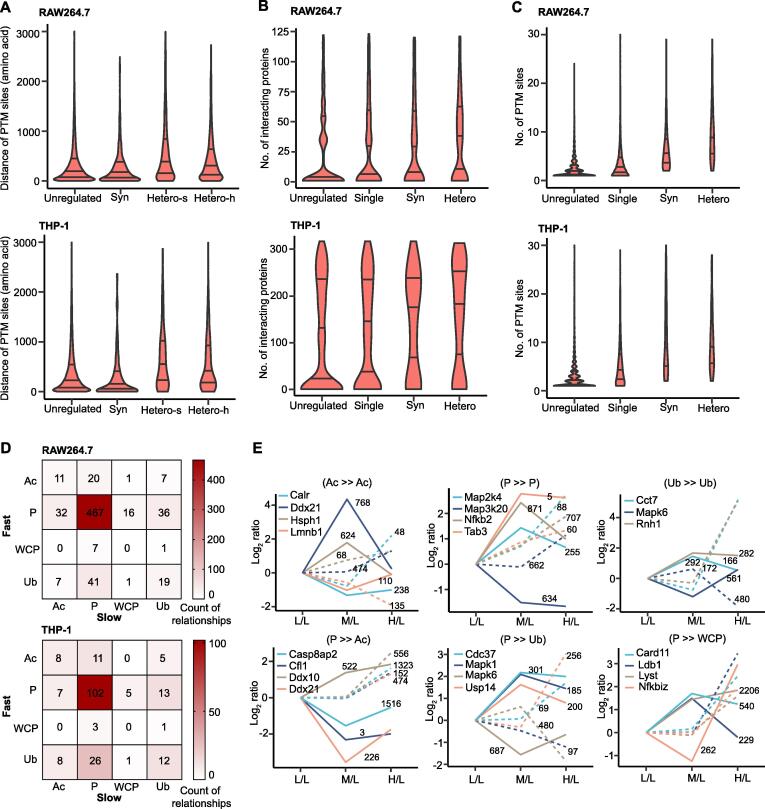
**Crosstalk between multiple PTM sites on the same protein** **A.** Distribution of the sequence distance between different PTM sites for proteins in the unregulated, Syn, Hetero-s, and Hetero-h groups. **B.** Distribution of the number of interacting proteins for proteins in the unregulated, Single, Syn, and Hetero groups. The number of interacting proteins was acquired from IntAct. **C.** Distribution of the number of identified PTM sites for each protein in unregulated, Single, Syn, and Hetero groups. In (A–C), the lower, median, and upper lines in each violin plot correspond to 25%, 50%, and 75%, respectively. **D.** Heatmap indicating all relationships between different proteomes including three types of PTM proteomes and WCP. **E.** Selected proteins in RAW264.7 cells belonging to the important combined PTM types listed in (D). The solid line represents a fast-regulated event and the dotted line represents a slow-regulated event. The number next to the line represents the positon of the PTM site on the corresponding protein. The PTM on the left of “≫” represents the slow-type PTM and the PTM on the right of “≫” represents the fast-type PTM. Hetero-s, synchronous PTM site in heterochronous protein; Hetero-h, heterochronous PTM site in heterochronous protein; Single, single-regulated protein.

We next analyzed the crosstalk between multiple PTM sites on the same protein. As shown in Figure 5A, the average sequence distance of PTM sites in synchronous proteins was shorter than that in the unregulated proteins. We then further measured the sequence distance of PTM sites in heterochronous proteins into two ways: one only measured the distance between the synchronous PTM sites, while the other only measured the distance between heterochronous PTM sites. The average sequence distances of PTM sites measured in two ways in the heterochronous proteins were much longer than that in the unregulated proteins and synchronous proteins, and the sequence distance of the synchronous PTM sites in the heterochronous proteins was longer than that of the heterochronous PTM sites. After counting the numbers of interacting proteins in each group mentioned in Figure 3E, the numbers of interacting proteins in descending order were heterochronous proteins, synchronous proteins, single-regulated proteins, and unregulated proteins (Figure 5B; Table S3). Additionally, the numbers of total PTM sites in each protein followed the same order as the numbers of interacting proteins (Figure 5C). However, the M/L values of PTM sites in unregulated proteins, single-regulated proteins, synchronous proteins, and heterochronous proteins were undifferentiated (Figure S5A). We drew a table to present the relationships of different PTMs from the fast and slow groups. Most of the fast-type acetylation sites were more likely to coexist with the slow-type phosphorylation and acetylation sites, while most of the fast-type ubiquitination sites coexisted with the slow-type phosphorylation and ubiquitination sites. A striking observation from this table was that the intersection between the fast- and slow-type phosphorylation sites was particularly large (Figure 5D). These propensities of PTM sites between fast and slow groups have examples in the immune response. In the TLR4 pathway, proteins such as Map2k4, Map3k20, Nfkb2, Tab3, and NFKBIE contained both fast- and slow-type phosphorylation sites. Moreover, Mapk6 possessed both a fast-type ubiquitination site and a slow-type ubiquitination site, while TAB2 contained both a fast-type phosphorylation site and a slow-type ubiquitination site (Figure 5E, Figure S5B).

### LPS induced both degradative and non-degradative ubiquitination

**Figure 6:**
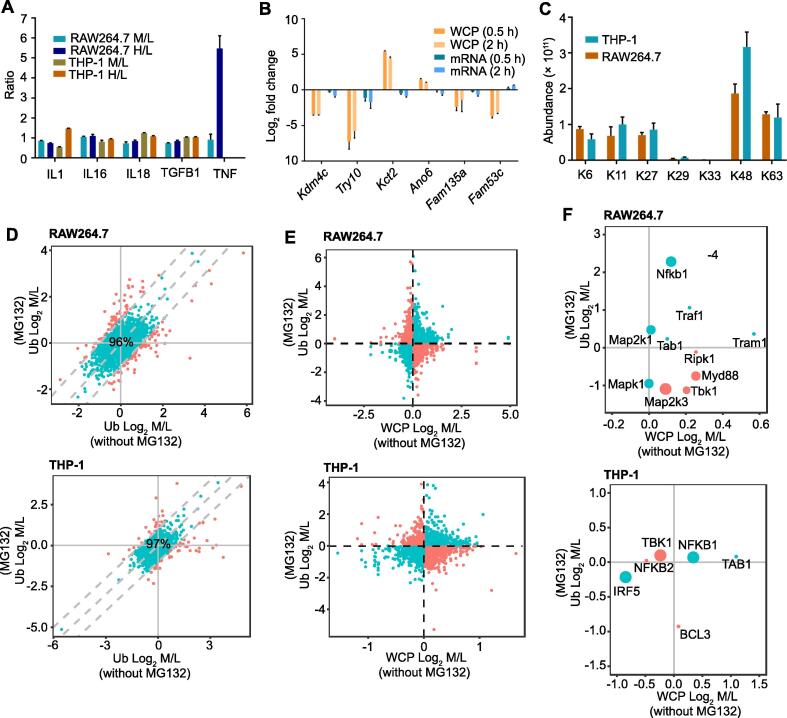
**Degradative and non-degradative ubiquitination both exist in the LPS-stimulated ubiquitinome** **A.** WCP ratios of five selected inflammatory factors. **B.** The relative protein and mRNA levels (fold change related to 0 h) for six selected genes in RAW264.7 cells with LPS stimulation. **C.** Abundance of ubiquitin lysine sites quantified in the Ub of LPS-stimulated macrophages. In (A–C), dada are represented as mean ± SEM (*n* = 3). **D.** Comparison of the Log_2_ M/L values of the ubiquitination sites from the LPS-stimulated Ub of cells treated with or without MG132 for 2 h. Sites exhibiting Log_2_ M/L ≥ 1 in untreated and MG132-treated cells were considered dramatically affected by MG132 (highlighted in red). **E.** Comparison of the Log_2_ M/L values of ubiquitination sites from the LPS-stimulated WCP of cells without MG132 treatment and the Ub of cells treated with MG132. Sites showing the same changes in the two proteome datasets were predicted to be non-degradative ubiquitination sites (highlighted in cyan), while the remaining sites were degradative ubiquitination sites (highlighted in red). **F.** Proteins in the TLR4 pathway illustrate the classification in (E).

Ubiquitination usually results in two distinct outcomes: degradative and non-degradative processes. We thus integrated the WCP and ubiquitinome datasets acquired from cells treated with or without MG132 to distinguish the ubiquitination results after LPS stimulation. We first investigated whether the protein levels would be influenced by the mRNA levels in a short time. The mRNA levels of IL-1, IL-16, IL-18, TGFB1, and TNF are known to increase after LPS stimulation in 0.5 h [19]. However, the protein levels of these inflammatory cytokines did not change in either RAW264.7 or THP-1 cells after 0.5 h LPS stimulation (Figure 6A). We also performed a RT-qPCR analysis on a set of genes whose protein levels were significantly regulated in our WCP dataset, and found that their mRNA levels did not change obviously (Figure 6B). Together, these results suggest that the mRNA levels have little effect on the protein levels at 0.5 h after LPS stimulation.

By looking at the relative abundance of ubiquitin lysine sites, we found that the ubiquitination of K48 (usually resulting in degradation) was dominant, followed by the ubiquitination of K63 (usually not resulting in degradation) (Figure 6C). Additionally, the ubiquitination of K48 and K63 was relatively stable during LPS stimulation (Figure S6A). A closer inspection of the ubiquitinome and WCP revealed that some ubiquitination sites (4% in RAW264.7 cells and 3% in THP-1 cells) and proteins (1% in both RAW264.7 and THP-1 cells) in LPS-stimulated macrophages were dramatically affected by MG132 (Figure 6D, Figure S6B).

We next predicted the outcomes of ubiquitinated proteins, according to the hypothesis that, for degradative ubiquitination, the changes in ubiquitination are inversely proportional to the changes in protein levels (Figure 6E). We found that non-degradative ubiquitination was widespread after LPS stimulation, including Nfkb1, Mapk1, and Traf1 (Figure 6F). Notably, MG132 modulated LPS-induced inflammation (Figure S6C).

### New proteins and PTMs involved in inflammatory response

**Figure 7:**
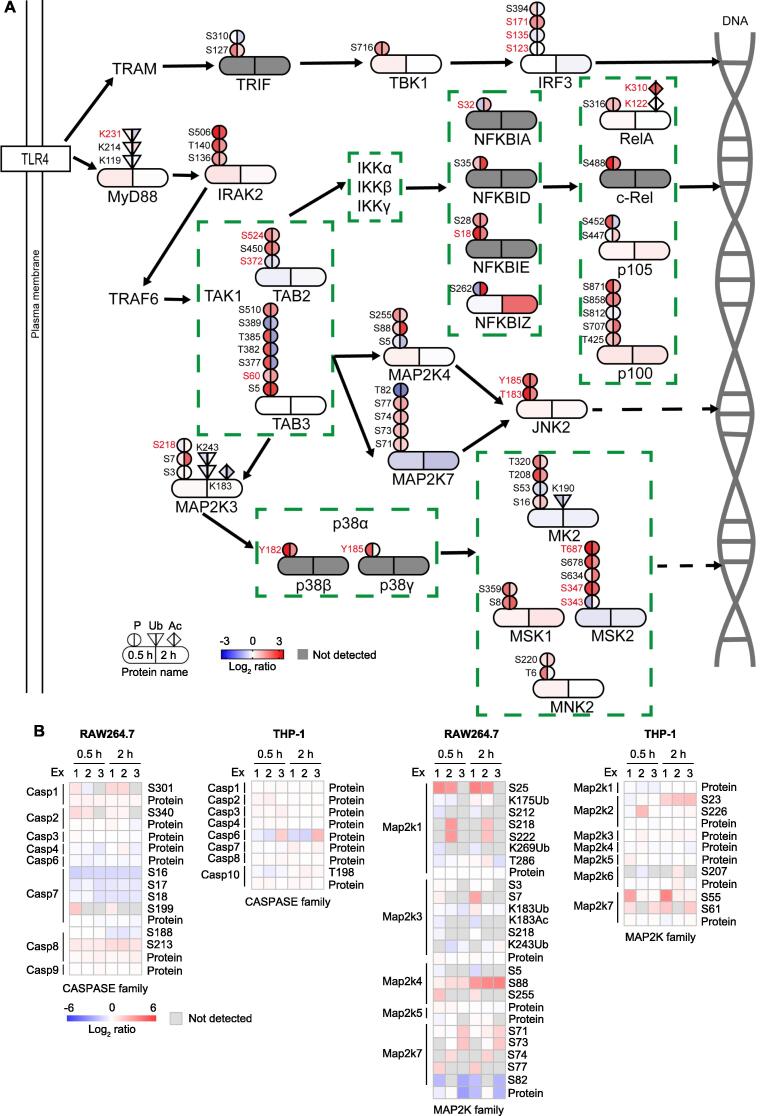
**New PTMs in the TLR4 signaling pathway involved in inflammatory response** **A.** Regulated proteins in the TLR4 pathway are grouped by function, and arrows indicate the direction of signal transduction. The colors on the left and right represent Log_2_ ratio of the indicated proteome datasets from cells stimulated with LPS for 0.5 h and 2 h, respectively. PTM sites with known functions based on UniProt and previous studies are colored in red [30], [31], [32], [33], [34], [35], [36], [37], [38], [39], [40]. **B.** Heatmap representation of the intensity of proteins and PTM sites involved in inflammatory signaling pathways detected in cells stimulated with LPS for 0.5 h and 2 h.

**Figure 8:**
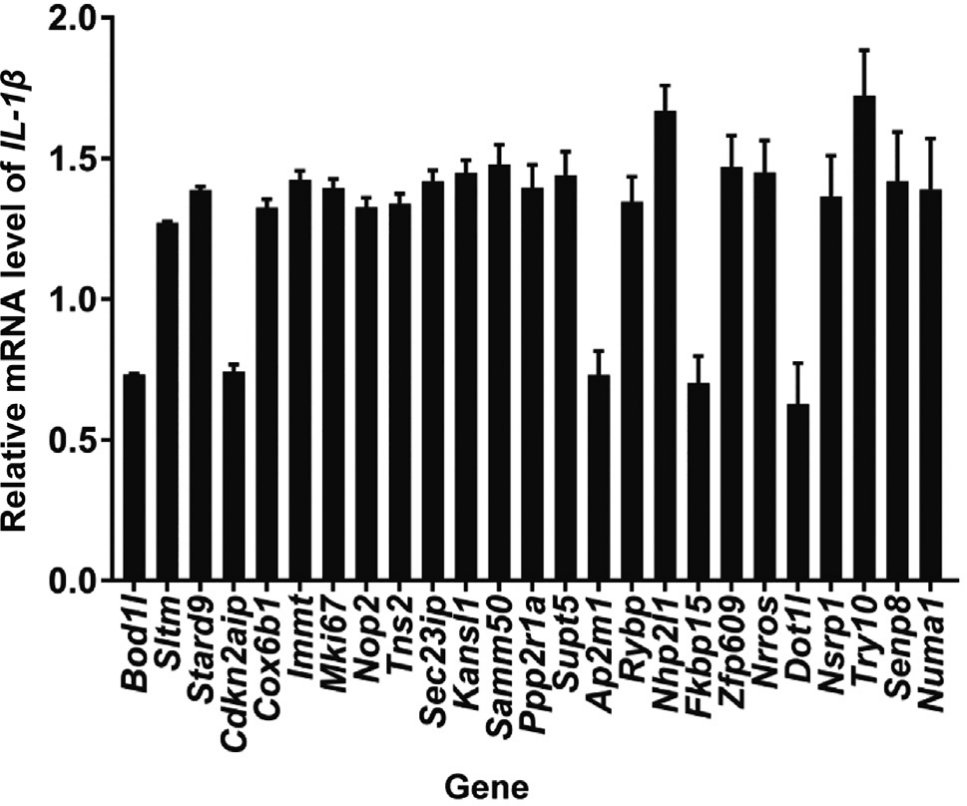
**RNAi screening for new proteins involved in inflammatory response** Bar chart showing the results of the RNAi screening for proteins containing apparently regulated PTM sites in mouse peritoneal macrophage cells. The relative mRNA level of *IL-1β* was generated by comparing the effect of a certain siRNA with its negative control siRNA. The bar chart was based on data obtained from two biological replicates, each containing three technical replicates. Error bar represents the standard error of the mean.

We integrated the WCP, acetylome, phosphoproteome, and ubiquitinome datasets to construct a network of regulated proteins with the PTM patterns in the MAPK and NF-κB signaling pathways. We also investigated the PTM patterns of proteins involved in major inflammatory signaling pathways activated by LPS stimulation. We mapped the acetylation, phosphorylation, and ubiquitination variations at different time points in this network. Twenty-two proteins (IRAK2, IRF3, TAB2, TAB3, TBK1, TRIF, MAPK9, MAPK11, MAPK12, MAP2K3, MAP2K4, MAP2K7, MAPKAPK2, MNK2, MSK1, MSK2, C-Rel, NFKB1, NFKB2, NFKBIE, NFKBID, and NFKBIZ) in this network exhibited up-regulated phosphorylation sites, while four proteins (TAB3, MAP2K7, MSK2, and NFKBIZ) displayed down-regulated phosphorylation sites. Besides, some of these proteins contained more than one regulated phosphorylation site. For example, TAB3 contained seven regulated phosphorylation sites, but only the phosphorylation on S60 has been well studied. Interestingly, TAB3 and MAP2K7 contained both up-regulated and down-regulated phosphorylation sites. By contrast, the acetylation and the ubiquitination of the proteins in this network were generally unregulated, except the acetylation on K310 in RelA. Nevertheless, two proteins (MyD88 and MK2) contained low levels (Log_2_ ratio ≥ 0.5 or ≤ −0.5) of regulated ubiquitination sites (Figure 7A). Furthermore, regulated PTM sites were widely found in several protein families associated with inflammation in both RAW264.7 and THP-1 cells, including the CASPASE family, MAPK family, MAP2K family, MAP3K family, INF-related family, TRAF family, and RIPK family (Figure 7B, Figure S7). To help identify new proteins involved in the inflammatory response, we selected 95 proteins that contained significantly regulated PTM sites but lacked studies on inflammation. We further studied these proteins using their corresponding siRNAs, and the results are displayed in Figure 8 and Table S4. Homology comparison of the regulated proteins identified in RAW264.7 and THP-1 cells revealed that the PTMs of 91 proteins were conserved between humans and mice (Table S5). All the PTM data obtained in this study were visualized in a PTM-inflammation website (http://ptm-inflammation.cn) (Figure S8).

## Discussion

Previous studies have reported LPS-induced proteome or phosphoproteome data in RAW264.7 and THP-1 cells [20], [21]. However, these studies primarily performed functional analyses of differentially modified proteins, and the main type of PTM they focused on was phosphorylation. In this study, we integrated WCP, acetylome, phosphoproteome, and ubiquitinome datasets to identify LPS stimulation-dependent PTM events in macrophages. These data provide insights into the crosstalk between PTMs during the inflammatory response. Although we did not validate the PTM data by biochemical approaches, our results were consistent with previous findings, such as the phosphorylation on Y182 of P38β, the phosphorylation on Y185 of P38γ, and the phosphorylation on S18 of NFKBIE (Figure 7A). Additionally, we performed RNAi screening in mouse peritoneal macrophage cells. As expected, the inflammatory response was affected by reducing the proteins whose PTMs were significantly altered in cell lines.

Within our datasets, phosphorylation had a significant advantage in terms of the number of quantified sites. For all PTM proteome data from both macrophage cell lines (except the phosphoproteome data from RAW264.7 cells), the proportion of up-regulated PTM sites exceeded the proportion of down-regulated ones; as the duration of LPS stimulation increased, the proportion of up-regulated PTM sites increased, while the proportion of down-regulated sites decreased. We suggest that the faster reaction rate of phosphorylation leads to the different phosphorylation trends as we observed in RAW264.7 cells. Additionally, the PTM properties of different cells were slightly different, and different PTMs usually occurred in the proteins of distinct functional groups. However, the WCP showed obvious randomness in functional groups, suggesting that the LPS-induced signal transduction is less dependent on the changes in protein levels.

In our study, a large number of proteins contained at least one type of regulated PTM. Within one protein, crosstalk events usually occur among nearby PTM sites, so the sequence distances between crosstalk pairs are shorter than average [22]. Therefore, the regulated PTM sites in synchronously proteins are more likely to be involved in crosstalk due to their shorter sequence distance than the PTM sites in unregulated proteins. In contrast, the sequence distance for both synchronous and heterochronous PTM sites in heterochronous protein was longer than the PTM sites in unregulated proteins. Additionally, more interacting partners were observed for heterochronous proteins than synchronous proteins. Interestingly, the number of PTM sites was proportional to the number of interacting proteins. Overall, we concluded that the regulated PTM sites in heterochronous proteins are more likely to have crosstalk with PTM sites in the corresponding interacting proteins. It can thus be suggested that the PTM sites participating in the crosstalk between proteins have longer sequence distance because they tend to locate in different domains in a protein.

MG132, which inhibits the degradation of ubiquitinated proteins, has been widely used to study the molecular mechanism of inflammation [23], [24], [25]. However, MG132 can directly influence the ubiquitination of certain proteins or indirectly influence ubiquitination by inhibiting the inflammatory response of macrophages. Thus, it is difficult to distinguish degradative and non-degradative ubiquitination by comparing the outcomes in cells treated with or without MG132. Therefore, we proposed a more reliable method to determine the levels of degradative and non-degradative ubiquitination according to whether the changes in protein levels were consistent with the changes in ubiquitination levels after the addition of MG132. Using this technique, we discovered that both degradative and non-degradative ubiquitination are prevalent in the immune response to LPS stimulation. The premise of this method is to limit the reaction time, so that the effect of gene expression on protein levels can be ignored. Complementarily, the integrated analysis of ubiquitinome, WCP, and transcriptome data solves this problem when the effect of gene expression on protein levels cannot be ignored [26]. Although these methods eliminate the negative effects of MG132, a specific inhibitor for deubiquitination of a specific protein of interest is preferred.

In conclusion, this study provides high-quality proteome datasets of multiple PTMs from macrophage cell lines stimulated by LPS. These datasets are a valuable resource for screening new molecules involved in the inflammatory response. Furthermore, the newly identified PTM sites in known inflammation-related proteins could help discover the underlying molecular mechanisms of the inflammatory response. Importantly, our in-depth analyses have identified the interrelationships among the most common PTMs and pave the way for future studies aiming to determine the relationships among different PTMs.

## Materials and methods

### Reagents

MG-132 (Catalog No. S2619, Selleck, Houston, TX), PR-619 (Catalog No. S7130, Selleck), trypsin (Catalog No. BELT001, Beierli, Tianjin, China), PTMScan Ubiquitin Remnant Motif Kit (Catalog No. 5562, Cell Signaling Technology, Bossdun, MA), PTMScan Acetyl-Lysine Motif Kit (Catalog No. 3416, Cell Signaling Technology), PTMScan Phospho-Tyrosine Rabbit mAb Kit (Catalog No. 8803, Cell Signaling Technology), High-Select Fe-NTA Phosphopeptide Enrichment Kit (Catalog No. A32992, ThermoFisher Scientific, Waltham, MA), High-Select TiO_2_ Phosphopeptide Enrichment Kit (Catalog No. A32993, ThermoFisher Scientific), Protease and Phosphatase Inhibitor Cocktail (Catalog No. P1049, Beyotime, Shanghai, China), DMEM for SILAC (Catalog No. 88364, Gibco, Waltham, MA), Dialyzed Fetal Bovine Serum (Catalog No. 30067334, Gibco), L-lysine-^13^C_6_-^15^N_2_ (Catalog No. 88209, Gibco), L-arginine-^13^C_6_-^15^N_4_ (Catalog No. 89990, Gibco), L-lysine-4,4,5,5-D4 (Catalog No. 88438, Gibco), L-arginine-^13^C_6_ (Catalog No. 88433, Gibco), and RPMI 1640 medium for SILAC (Catalog No. 88365, Gibco).

### Cell culture and sample preparation

RAW264.7 and THP-1 cells were cultured in DMEM and RPMI 1640 medium, respectively, both without lysine and arginine but with excess L-proline. Subsequently, cells were grown in light media (supplemented with 100 mg/l L-lysine and 100 mg/l L-arginine), medium media (supplemented with 100 mg/l L-lysine-4,4,5,5-D4 and 100 mg/l L-arginine-^13^C_6_), or heavy media (supplemented with 100 mg/l L-lysine-^13^C_6_-^15^N_2_ and 100 mg/l L-arginine-^13^C_6_-^15^N_4_), and then activated with 100 ng/ml LPS for 0 h, 0.5 h, and 2 h, respectively [19]. Before the follow-up experiments, we detected the SILAC experiments with a labeling efficiency of over 98%. For MG132 treatments, all SILAC-labeled cells were treated with 5 µM MG132 for 2 h, and then 1–3 × 10^7^ cells were harvested for each experiment and lysed with freshly prepared lysis buffer (8 M urea, 150 mM NaCl, 50 mM Tris-HCl pH 8.0, 1× phosphorylase inhibitor, 1× EDTA, 50 µM PR-619, and 1× protease inhibitor). Protein concentrations were estimated using the BCA kit, and lysates from each cell line treated with light, medium, and heavy media were combined in a 1:1:1 ratio. The combined lysates were reduced with 10 mM dithiothreitol (DTT), carbamidomethylated with 30 mM iodoacetamide (IAA), and digested overnight with trypsin. Peptides were acidified with trifluoroacetic acid (TFA), desalted with preconditioned C18 Sep-Pak SPE cartridges, and lyophilized for 48 h with a vacuum lyophilizer (LABCONCO, Fort Scott, KS). Peritoneal macrophages were acquired from 6–8-week-old male C57/BL6 mice.

### Enrichment of ubiquitinated, acetylated, and phosphorylated peptides

For proteomics analyses of acetylation, p-Tyr, and ubiquitination, lyophilized peptides were resuspended in ice-cold 1× IAP buffer and incubated with corresponding cross-linked antibody beads for 2 h at 4 °C with end-over-end rotation. Beads were softly washed three times with ice-cold 1× IAP buffer and twice with ice-cold MS-quality water. Modified peptides were eluted twice by 50 µl 0.15% TFA. For TiO_2_ and Fe-NTA methods, lyophilized peptides were treated as described in the protocols provided with the corresponding kits. The enriched peptides were quickly dried by vacuum centrifugation and divided into eight fractions using HPRP. All peptides were desalted using homemade stage tip chromatography and dried by vacuum centrifugation before the MS analysis.

### Liquid chromatography–tandem mass spectrometry analysis

All peptide samples were resuspended in 2% acetonitrile (ACN) and 0.1% formic acid (FA). Then, peptides were separated by nano-liquid chromatography–tandem mass spectrometry (nanoLC–MS/MS) using an UltiMate 3000 RSLCnano system (ThermoFisher Scientific) and analyzed using Q Exactive HF-X (ThermoFisher Scientific). Gradient elution was performed at 32 °C for over 120 min using a gradient of 3%–80% ACN in 0.1% FA. The MS spectra of WCP, ubiquitinome, TiO_2_, and Fe-NTA were acquired at a resolution of 120,000 with a mass range of 300–1500 *m/z* and an automatic gain control (AGC) target of 3E6, while the MS spectra of acetylome and p-Tyr were acquired at a resolution of 60,000. The MS2 spectra were acquired at a resolution of 30,000, and higher energy collision induced dissociation (HCD) fragmentation was performed with a collision energy of approximately 27% normalized collisional energy (NCE). The isolation window was set to 1.0 *m/z* and the dynamic exclusion window was set to 30 s.

### Protein identification and quantification

MaxQuant (version 1.6.2.10) was used for protein identification and quantification. The human and mouse UniProtKB databases were utilized as the search databases. The variable modifications of the acetylome, phosphoproteome, and ubiquitinome included oxidation (M), acetyl (protein N-term), and corresponding PTMs. Carbamidomethyl (C) was set as the fixed modification. The maximum number of modifications for a peptide was set to 5. Trypsin was set as the digestion enzyme, and the maximum number of missed cleavage sites was set to 2. Additionally, we used 20 ppm as the ion tolerance in the first search and 4.5 ppm as the ion tolerance in the main search. Both peptide and protein identifications were performed at false discovery rate (FDR) < 1%. The default parameters of MaxQuant were adopted if not described above.

### RT-qPCR and RNAi screening

RAW264.7 cells were treated with 100 ng/ml LPS, and then RNA was isolated using an RNA extraction kit (Catalog No. 220011, Feijie, Shanghai, China) according to the manufacturer’s protocol. The cDNA templates were synthesized using an RNA reverse transcription kit (Takara, Tokyo, Japan). RT-qPCR was performed using TB Green Premix Ex Taq II (Takara). The siRNAs were purchased from Dharmacon and transfected into mouse peritoneal macrophage cells using the DharmaFECT 1 siRNA Transfection Reagent.

### Functional annotation enrichment analysis

Proteins containing PTM sites with a fold change ≥ 2 were selected for functional annotation enrichment analysis using WebGestalt 2019 [27] to identify the pathways in which different PTMs were enriched in cells stimulated with LPS based on the PTM proteome data. We chose the top three categories of the forty categories visualized in the report for each cell line and each time point. The heatmap was constructed using the pheatmap package in R.

### Identification of PTM consensus motifs

We analyzed sequences within −15 to +15 amino acids of the quantified PTM sites (the PTM site was located at position 0) to explore the consensus sequence of amino acids surrounding each PTM. iceLogo was used to generate the amino acid sequence diagram [28].

### Statistical analysis

The R framework (version 3.5.1) and GraphPad Prism (version 7) software were used to perform all statistical analyses of the bioinformatics data. All the proteome datasets were generated in biological triplicates. The RT-qPCR analysis of mRNA abundance was performed in biological triplicates. The RNAi screening experiment was performed using biological duplicates. The table listing the PTM sites was filtered to remove the entries with a localization probability less than 90%. The PTM sites with a membership greater than 0.6 were classified into the corresponding category using the fuzzy c-means method. The interaction network only retained the interactions with a STRING database score greater than 0.8.

## Ethical statement

This study was approved by the ethics committees of the First Affiliated Hospital of Zhejiang University, China.

## Data availability

The mass spectrometry proteomics data have been deposited in the ProteomeXchange Consortium via the iProX partner repository [29] (ProteomeXchange: PXD015527), and are publicly accessible at https://www.iprox.cn/. The PTM-inflammation website is freely accessible at http://ptm-inflammation.cn.

## CRediT author statement

**Feiyang Ji:** Conceptualization, Methodology, Formal analysis, Investigation, Writing - original draft, Writing - review & editing, Visualization. **Menghao Zhou:** Methodology, Investigation, Writing - original draft, Writing - review & editing. **Huihui Zhu:** Investigation. **Zhengyi Jiang:** Methodology, Investigation. **Qirui Li:** Investigation. **Xiaoxi Ouyang:** Investigation. **Yiming Lv:** Formal analysis, Writing - review & editing, Visualization. **Sainan Zhang:** Investigation. **Tian Wu:** Formal analysis. **Lanjuan Li:** Conceptualization, Resources, Data curation, Supervision, Funding acquisition. All authors have read and approved the final manuscript.

## Competing interests

The authors have declared no competing interests.

## Supplementary material

Supplementary Figure S1 **Reproducible integrative proteomics analysis of LPS-stimulated macrophages A.** Intersection of phosphosites quantified with the three methods used to analyze the P. **B.** Overlap between proteins quantified in the WCP and diGly sites quantified in the Ub in “MG132, presence of MG132” and “None, absence of MG132” groups. **C.** Number of PTM sites that were quantified in each of 1, 2 or 3 Ac, Fe-NTA, P-Tyr and Ub experiments in two cell types. **D.** Pearson’s correlation plots for two “Ex, representative experiments” from WCP, Fe-NTA, TiO2, Ac, and P-Tyr in RAW264.7 and THP-1 cells. The inserted table shows Pearson’s correlation coefficients for all three biological duplicates.

Supplementary Figure S2 **Properties and differences among various types of proteomics approaches A.** Distribution of regulated acetylated, phosphorylated, and ubiquitinated sites per protein in macrophages stimulated with LPS for 0.5 hours and 2 hours. **B.** Changes in integrative proteomics data obtained from LPS-treated THP-1 cells over time. Regulated proteins and PTM sites were clustered into the six indicated categories using the fuzzy c-means method. **C.** The distribution of categories of regulated sites in the corresponding proteins identified using integrative proteomics. The six categories in B were combined into two categories according to the speed of change.

Supplementary Figure S3 **PTM crosstalk between different proteins A.** Density gradient diagram of the Log_2_ ratio of proteins and PTM sites in the different proteomes of THP-1 cells. Carmine and cyan represent cells stimulated with LPS for 0.5 hours and 2 hours, respectively. Carmine and cyan numbers on the left and right represent the number and percentage of regulated proteins and PTM sites in the two time points, respectively. **B.** Heatmap representation of the Log_2_ (M/L) of the abundance of proteins quantified in THP-1 cells using the WCP and all PTM proteomics methods. Only proteins with a Log_2_ (M/L) value ≥ 1 or ≤ −1 are shown, and the color of proteins identified using PTM proteomics indicate the mean Log_2_ (M/L) ratio of all PTM sites in the protein. **C.** The iceLogo plots show the difference of amino acid frequency at positions flanking the PTM sites for LPS-regulated PTM sites compared to unregulated PTM sites with a *P* value ≤ 0.05 in THP-1 cells. **D.** Interaction network for proteins with regulated PTM sites in THP-1 cells. Blue dots indicate the proteins with no interacting partners, while red dots indicate the interacting proteins. The size of the dot indicates the number of regulated PTM sites.

Supplementary Figure S4 **Annotation enrichment analysis of regulated proteins** Annotation enrichment analysis of proteins with regulated expression level (top left panel), proteins with regulated acetylation sites (bottom left panel), proteins with regulated phosphorylated sites (top right panel) and proteins with regulated ubiquitinated sites (bottom right panel). Only the terms with the top three −Log10 (FDR) values in each of the following categories for all time points and both cell lines are shown: “BP, biological processes”, “CC, cellular compartments”, “KEGG, pathways”, and “MF, molecular functions”.

Supplementary Figure S5 **PTM crosstalk between multiple sites on the same protein A.** Distribution of the ratio of all PTM sites identified in proteins in the unreg, single, syn, and hetero groups. The lower, median, and upper lines in each violin plot correspond to 25%, 50%, and 75%, respectively. **B.** Selected regulated proteins in Thp1 cells belonging to the corresponding category listed on top of each panel. The solid line represents a ‘fast’ regulated event and the dotted line represents a ‘slow’ regulated event. The number next to the line represents the site of PTM on the corresponding protein. The PTM in the left of “>>” is ‘slow’ PTM and the PTM in the right of “>>” is ‘fast’ PTM.

Supplementary Figure S6 **Integrative proteomics reveals a prevalence of both degradative and non-degradative ubiquitylation A.** The ratio of the abundance of ubiquitin lysine sites quantified in the Ub of Raw and Thp1 cells following LPS stimulation with the presence of MG132. **B.** Comparison of Log2 (M/L) values of proteins abundance in the WCP of LPS-stimulated cells treated with or without MG132 for 2 hours. Proteins that exhibited a ≥ 1 Log2 (M/L) difference in untreated and MG132-treated cells were considered dramatically affected by MG132 (carmine). **C.** The relative mRNA levels of IL-1β and IL-6 were generated from the comparison of a certain group with the “NC, negative control”. One representative containing three technical replicates out of two independent experiments is shown. MG132 (2 µM) and LPS were added at the same time. Error bars represent the standard error of the mean and statistical significance was determined by t-test (**P* ≤ 0.05; ***P* ≤ 0.01; ****P* ≤ 0.001).

Supplementary Figure S7 **Regulated proteins involved in inflammatory signaling pathways after cells were stimulated with LPS** The diagram shows the intensity of signals for proteins and PTM sites involved in IRF (A, F), MAP3K (B, G), MAPK (C, H), RIPK (D, I), and TRAF (E, J) inflammatory signaling pathways identified in cells stimulated with LPS for 0.5 hours and 2 hours.

Supplementary Figure S8 **A database of PTM in inflammation** Map2k3 served as an example of the PTM-inflammation website (http://ptm-inflammation.cn). The figure shows basic information about the protein, such as the sub-cellular location, modification type, UniProt description, sequence, etc. It also includes a visualization of post-translational data and a table providing detailed data.

Supplementary Table S1 **WCP and PTM data of RAW264.7 and THP-1 cells**

Supplementary Table S2 **Interacting proteins containing PTM crosstalk across proteins**

Supplementary Table S3 **Time series analysis of PTM data**

Supplementary Table S4 **siRNA screening results and siRNA sequences**

Supplementary Table S5 **Conserved proteins between RAW264.7 and Thp1 cells**

## References

[1] Geissmann F., Manz M.G., Jung S., Sieweke M.H., Merad M., Ley K. (2010). Development of monocytes, macrophages, and dendritic cells. Science.

[2] Murray P.J., Wynn T.A. (2011). Protective and pathogenic functions of macrophage subsets. Nat Rev Immunol.

[3] Liu J., Qian C., Cao X. (2016). Post-translational modification control of innate immunity. Immunity.

[4] Karin M., Ben-Neriah Y. (2000). Phosphorylation meets ubiquitination: the control of NF-κB activity. Annu Rev Immunol.

[5] Arthur J.S.C., Ley S.C. (2013). Mitogen-activated protein kinases in innate immunity. Nat Rev Immunol.

[6] Liu S., Cai X., Wu J., Cong Q., Chen X., Li T. (2015). Phosphorylation of innate immune adaptor proteins MAVS, STING, and TRIF induces IRF3 activation. Science.

[7] Jiang X., Chen Z.J. (2012). The role of ubiquitylation in immune defence and pathogen evasion. Nat Rev Immunol.

[8] Ikeda F. (2015). Linear ubiquitination signals in adaptive immune responses. Immunol Rev.

[9] Komander D., Rape M. (2012). The ubiquitin code. Annu Rev Biochem.

[10] Skaug B., Jiang X., Chen Z.J. (2009). The role of ubiquitin in NF-κB regulatory pathways. Annu Rev Biochem.

[11] Haberland M., Montgomery R.L., Olson E.N. (2009). The many roles of histone deacetylases in development and physiology: implications for disease and therapy. Nat Rev Genet.

[12] Kanno Y., Vahedi G., Hirahara K., Singleton K., O'Shea J.J. (2012). Transcriptional and epigenetic control of T helper cell specification: molecular mechanisms underlying commitment and plasticity. Annu Rev Immunol.

[13] Li X., Zhang Q., Ding Y., Liu Y., Zhao D., Zhao K. (2016). Methyltransferase Dnmt3a upregulates HDAC9 to deacetylate the kinase TBK1 for activation of antiviral innate immunity. Nat Immunol.

[14] Mowen K.A., David M. (2014). Unconventional post-translational modifications in immunological signaling. Nat Immunol.

[15] Cohen P. (2014). Immune diseases caused by mutations in kinases and components of the ubiquitin system. Nat Immunol.

[16] Huai W., Liu X., Wang C., Zhang Y., Chen X., Chen X. (2019). KAT8 selectively inhibits antiviral immunity by acetylating IRF3. J Exp Med.

[17] Venne A.S., Kollipara L., Zahedi R.P. (2014). The next level of complexity: crosstalk of posttranslational modifications. Proteomics.

[18] Ong S.E., Blagoev B., Kratchmarova I., Kristensen D.B., Steen H., Pandey A. (2002). Stable isotope labeling by amino acids in cell culture, SILAC, as a simple and accurate approach to expression proteomics. Mol Cell Proteomics.

[19] Zhao X.B., Ji F.Y., Li H.R., Zhu H.H., Zhao Z.Z., Ling J. (2020). P22077 inhibits LPS-induced inflammatory response by promoting K48-linked ubiquitination and degradation of TRAF6. Aging (Albany NY).

[20] Luo Y., Jiang Q., Zhu Z., Sattar H., Wu J., Huang W. (2020). Phosphoproteomics and proteomics reveal metabolism as a key node in LPS-induced acute inflammation in RAW264.7. Inflammation.

[21] Meijer K., Weening D., de Vries M.P., Priebe M.G., Vonk R.J., Roelofsen H. (2015). Quantitative proteomics analyses of activation states of human THP-1 macrophages. J Proteomics.

[22] Liu H.F., Liu R. (2020). Structure-based prediction of post-translational modification cross-talk within proteins using complementary residue- and residue pair-based features. Brief Bioinform.

[23] Yin Q., Han T., Fang B., Zhang G., Zhang C., Roberts E.R. (2019). K27-linked ubiquitination of BRAF by ITCH engages cytokine response to maintain MEK-ERK signaling. Nat Commun.

[24] Annibaldi A., Wicky John S., Vanden Berghe T., Swatek K.N., Ruan J., Liccardi G. (2018). Ubiquitin-mediated regulation of RIPK1 kinase activity independent of IKK and MK2. Mol Cell.

[25] Ohtake F., Saeki Y., Ishido S., Kanno J., Tanaka K. (2016). The K48–K63 branched ubiquitin chain regulates NF-κB signaling. Mol Cell.

[26] Dybas J.M., O'Leary C.E., Ding H., Spruce L.A., Seeholzer S.H., Oliver P.M. (2019). Integrative proteomics reveals an increase in non-degradative ubiquitylation in activated CD4^+^ T cells. Nat Immunol.

[27] Liao Y., Wang J., Jaehnig E.J., Shi Z., Zhang B. (2019). WebGestalt 2019: gene set analysis toolkit with revamped UIs and APIs. Nucleic Acids Res.

[28] Colaert N., Helsens K., Martens L., Vandekerckhove J., Gevaert K. (2009). Improved visualization of protein consensus sequences by iceLogo. Nat Methods.

[29] Ma J., Chen T., Wu S., Yang C., Bai M., Shu K. (2019). iProX: an integrated proteome resource. Nucleic Acids Res.

[30] Fleming Y., Armstrong C.G., Morrice N., Paterson A., Goedert M., Cohen P. (2000). Synergistic activation of stress-activated protein kinase 1/c-Jun N-terminal kinase (SAPK1/JNK) isoforms by mitogen-activated protein kinase kinase 4 (MKK4) and MKK7. Biochem Soc.

[31] Fox T., Fitzgibbon M.J., Fleming M.A., Hsiao H.M., Brummel C.L., Su M.S.S. (1999). Kinetic mechanism and ATP-binding site reactivity of p38Q MAP kinase. FEBS Lett.

[32] Tomas-Zuber M., Mary J.L., Lesslauer W. (2000). Control sites of ribosomal S6 kinase B and persistent activation through tumor necrosis factor. J Biol Chem.

[33] Karpova A.Y., Trost M., Murray J.M., Cantley L.C., Howley P.M. (2002). Interferon regulatory factor-3 is an *in vivo* target of DNA-PK. Proc Natl Acad Sci U S A.

[34] Mahlknecht U., Will J., Varin A., Hoelzer D., Herbein G. (2004). Histone deacetylase 3, a class I histone deacetylase, suppresses MAPK11-mediated activating transcription factor-2 activation and represses *TNF* gene expression. J Immunol.

[35] Wang J.T., Doong S.L., Teng S.C., Lee C.P., Tsai C.H., Chen M.R. (2009). Epstein-Barr virus BGLF4 kinase suppresses the interferon regulatory factor 3 signaling pathway. J Virol.

[36] Moore T.C., Petro T.M. (2013). IRF3 and ERK MAP-kinases control nitric oxide production from macrophages in response to poly-I:C. FEBS Lett.

[37] Lee B.C., Miyata M., Lim J.H., Li J.D. (2016). Deubiquitinase CYLD acts as a negative regulator for bacterium NTHi-induced inflammation by suppressing K63-linked ubiquitination of MyD88. Proc Natl Acad Sci U S A.

[38] Zhang Q., Lenardo M.J., Baltimore D. (2017). 30 Years of NF-κB: a blossoming of relevance to human pathobiology. Cell.

[39] Thapa D., Nichols C., Bassi R., Martin E.D., Verma S., Conte M.R. (2018). TAB1-induced autoactivation of p38α mitogen-activated protein kinase is crucially dependent on threonine 185. Mol Cell Biol.

[40] Zhao Y., Mudge M.C., Soll J.M., Rodrigues R.B., Byrum A.K., Schwarzkopf E.A. (2018). OTUD4 is a phospho-activated K63 deubiquitinase that regulates MyD88-dependent signaling. Mol Cell.

