# Supplementary figures and images for: Integrative Proteomic Analysis of Multiple Posttranslational Modifications in Inflammatory Response
Source: Genomics Proteomics Bioinformatics. 2021 Mar 2;20(1):163–76. doi: 10.1016/j.gpb.2020.11.004 (PMC9510875; doi:10.1016/j.gpb.2020.11.004)

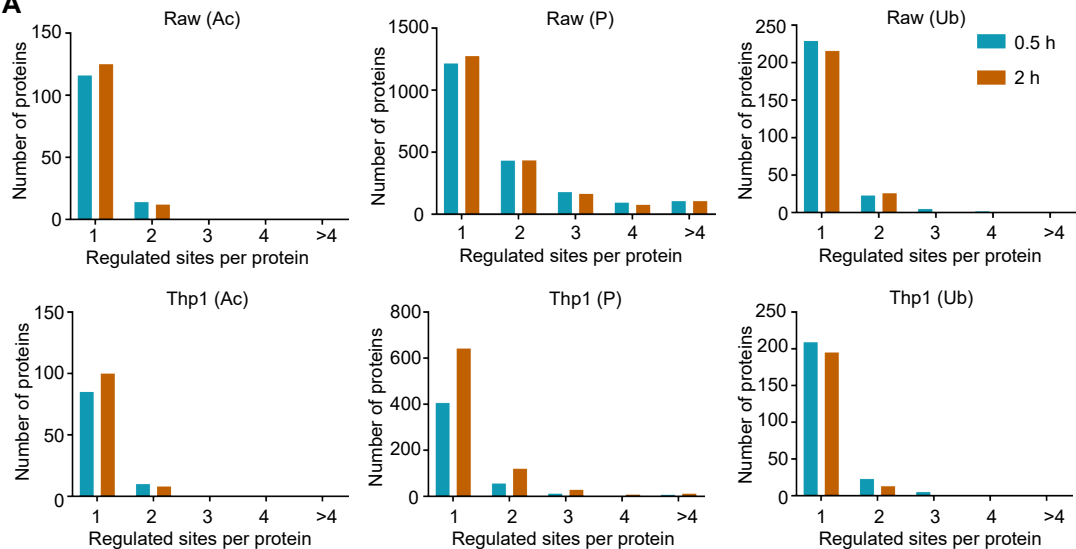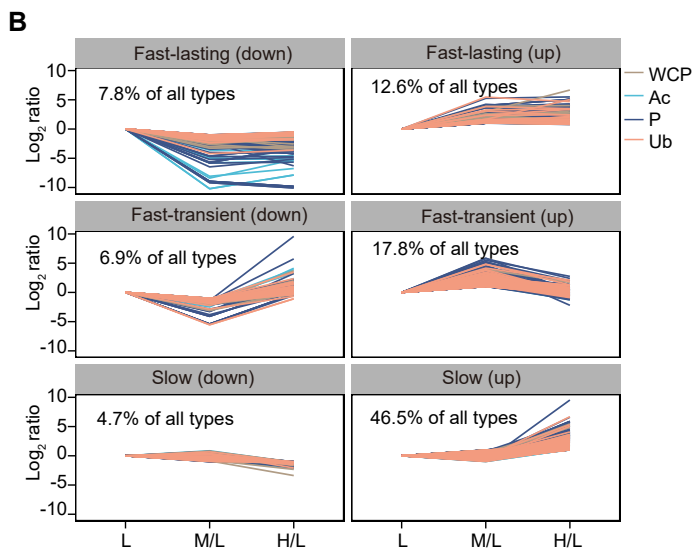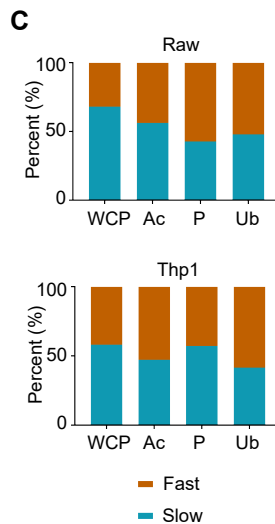

Supplement: Supplementary Figure S2 — Properties and differences among various types of proteomics approaches A. Distribution of regulated acetylated, phosphorylated, and ubiquitinated sites per protein in macrophages stimulated with LPS for 0.5 hours and 2 hours. B. Changes in integrative proteomics data obtained from LPS-treated THP-1 cells over time. Regulated proteins and PTM sites were clustered into the six indicated categories using the fuzzy c-means method. C. The distribution of categories of regulated sites in the corresponding proteins identified using integrative proteomics. The six categories in B were combined into two categories according to the speed of change. [file mmc2.pdf]

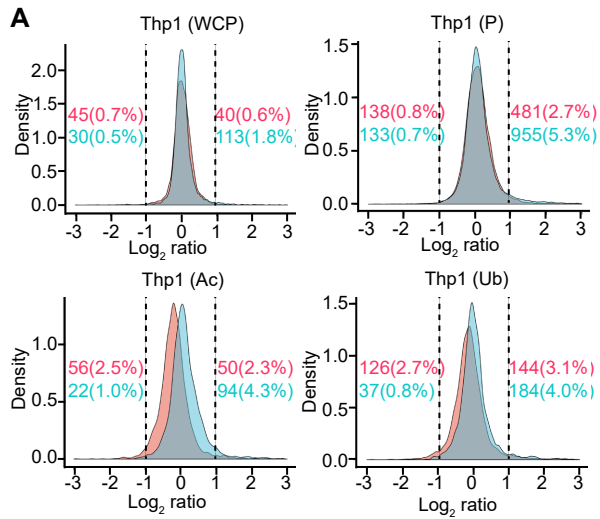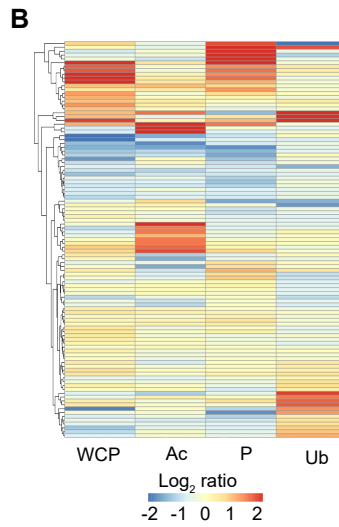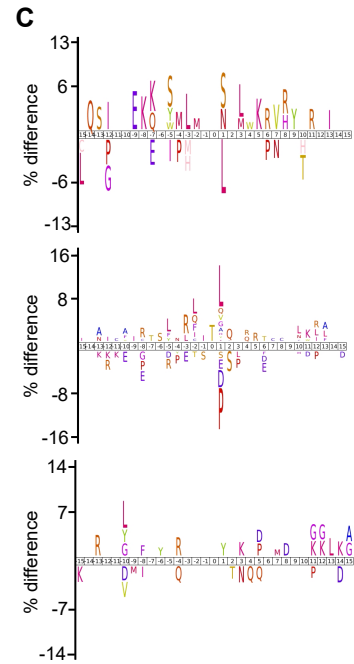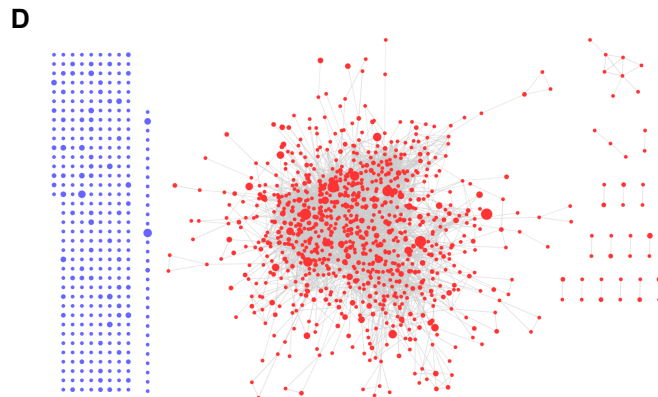

Supplement: Supplementary Figure S3 — PTM crosstalk between different proteins A. Density gradient diagram of the Log2 ratio of proteins and PTM sites in the different proteomes of THP-1 cells. Carmine and cyan represent cells stimulated with LPS for 0.5 hours and 2 hours, respectively. Carmine and cyan numbers on the left and right represent the number and percentage of regulated proteins and PTM sites in the two time points, respectively. B. Heatmap representation of the Log2 (M/L) of the abundance of proteins quantified in THP-1 cells using the WCP and all PTM proteomics methods. Only proteins with a Log2 (M/L) value ≥ 1 or ≤ −1 are shown, and the color of proteins identified using PTM proteomics indicate the mean Log2 (M/L) ratio of all PTM sites in the protein. C. The iceLogo plots show the difference of amino acid frequency at positions flanking the PTM sites for LPS-regulated PTM sites compared to unregulated PTM sites with a P value ≤ 0.05 in THP-1 cells. D. Interaction network for proteins with regulated PTM sites in THP-1 cells. Blue dots indicate the proteins with no interacting partners, while red dots indicate the interacting proteins. The size of the dot indicates the number of regulated PTM sites. [file mmc3.pdf]

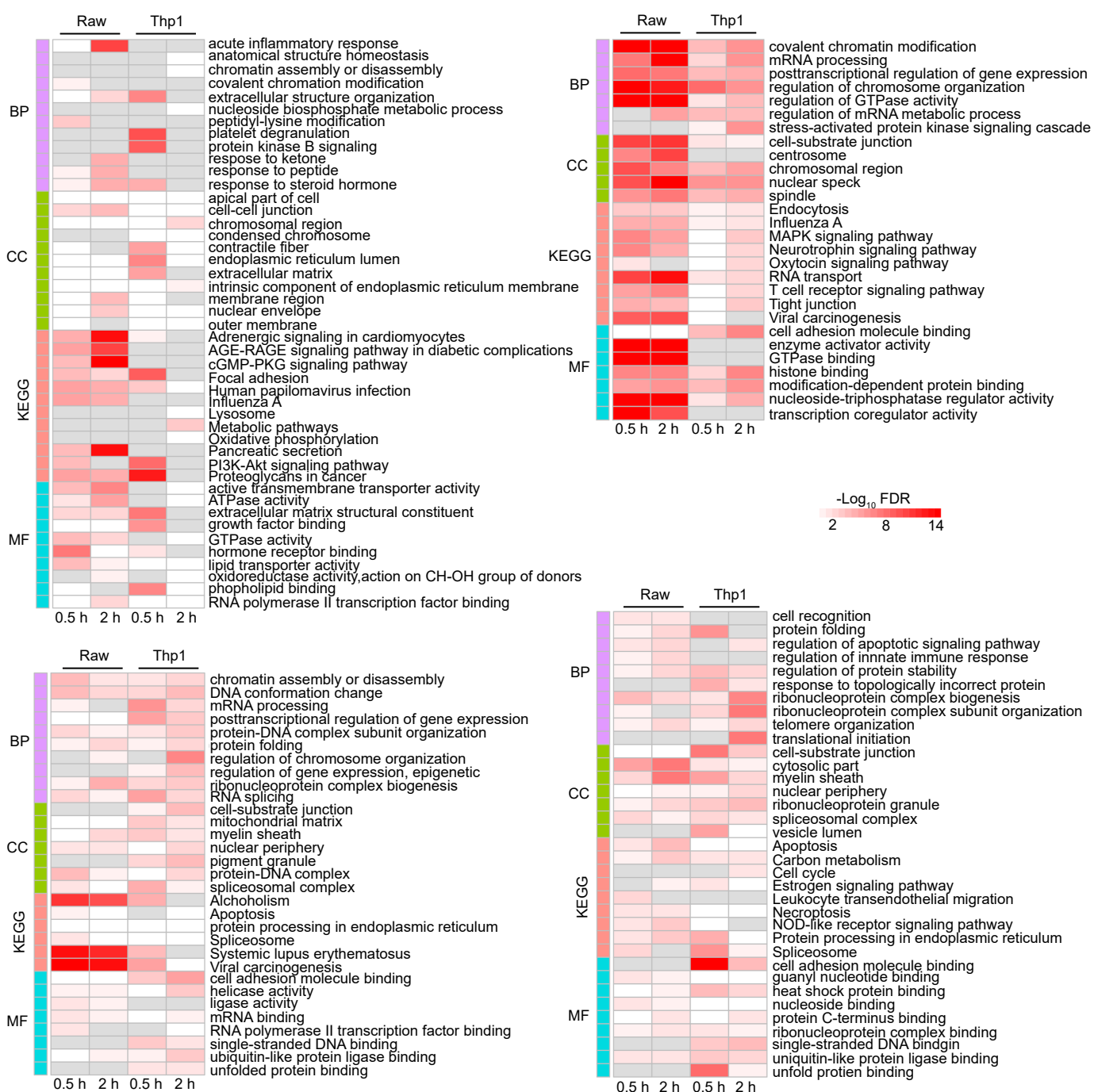

Supplement: Supplementary Figure S4 — Annotation enrichment analysis of regulated proteins Annotation enrichment analysis of proteins with regulated expression level (top left panel), proteins with regulated acetylation sites (bottom left panel), proteins with regulated phosphorylated sites (top right panel) and proteins with regulated ubiquitinated sites (bottom right panel). Only the terms with the top three −Log10 (FDR) values in each of the following categories for all time points and both cell lines are shown: “BP, biological processes”, “CC, cellular compartments”, “KEGG, pathways”, and “MF, molecular functions”. [file mmc4.pdf]

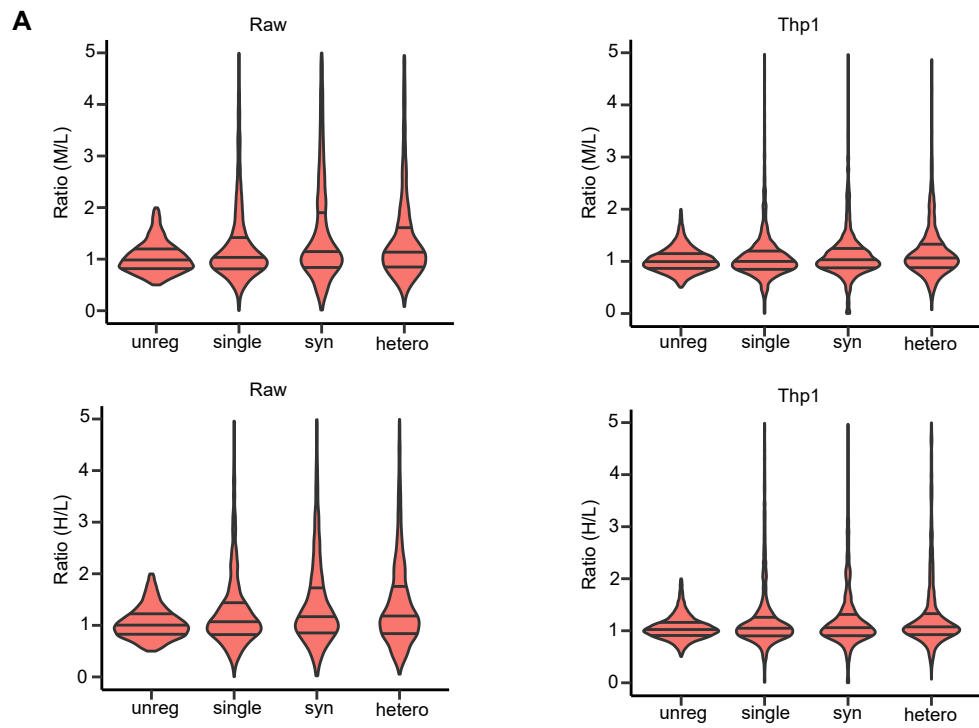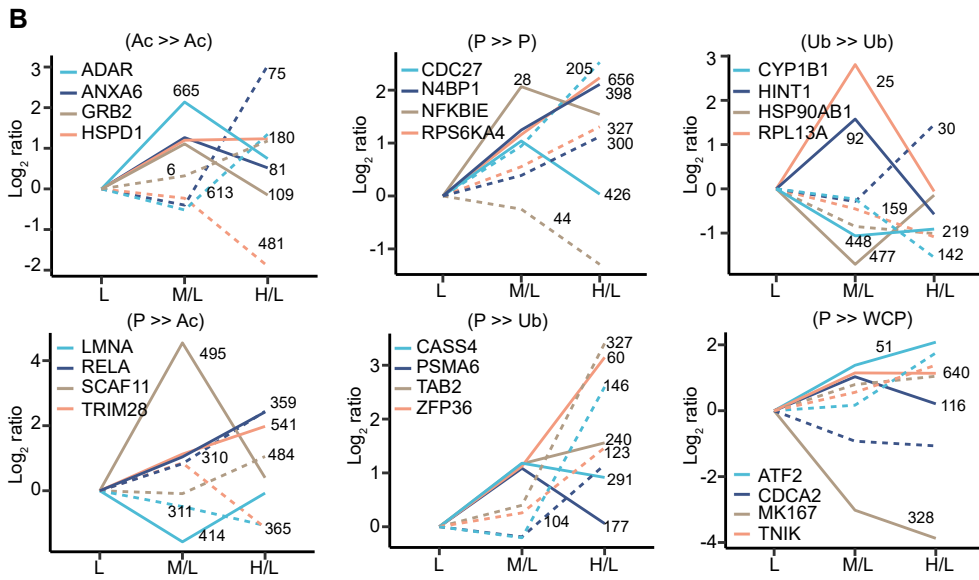

Supplement: Supplementary Figure S5 — PTM crosstalk between multiple sites on the same protein A. Distribution of the ratio of all PTM sites identified in proteins in the unreg, single, syn, and hetero groups. The lower, median, and upper lines in each violin plot correspond to 25%, 50%, and 75%, respectively. B. Selected regulated proteins in Thp1 cells belonging to the corresponding category listed on top of each panel. The solid line represents a ‘fast’ regulated event and the dotted line represents a ‘slow’ regulated event. The number next to the line represents the site of PTM on the corresponding protein. The PTM in the left of “>>” is ‘slow’ PTM and the PTM in the right of “>>” is ‘fast’ PTM. [file mmc5.pdf]

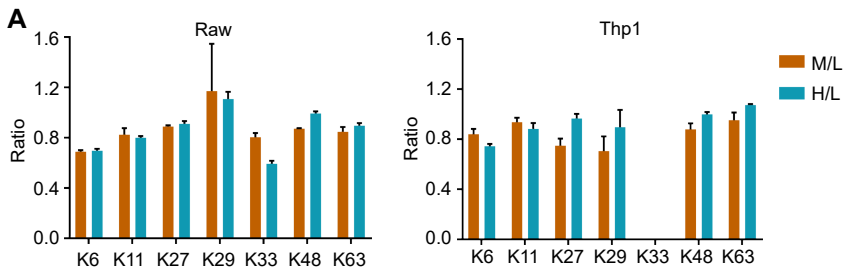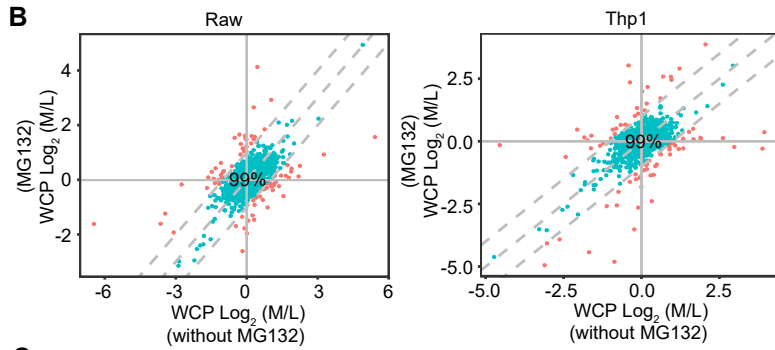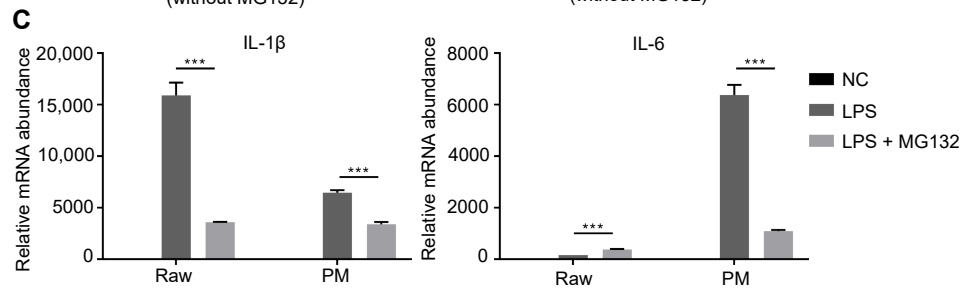

Supplement: Supplementary Figure S6 — Integrative proteomics reveals a prevalence of both degradative and non-degradative ubiquitylation A. The ratio of the abundance of ubiquitin lysine sites quantified in the Ub of Raw and Thp1 cells following LPS stimulation with the presence of MG132. B. Comparison of Log2 (M/L) values of proteins abundance in the WCP of LPS-stimulated cells treated with or without MG132 for 2 hours. Proteins that exhibited a ≥ 1 Log2 (M/L) difference in untreated and MG132-treated cells were considered dramatically affected by MG132 (carmine). C. The relative mRNA levels of IL-1β and IL-6 were generated from the comparison of a certain group with the “NC, negative control”. One representative containing three technical replicates out of two independent experiments is shown. MG132 (2 µM) and LPS were added at the same time. Error bars represent the standard error of the mean and statistical significance was determined by t-test (*P ≤ 0.05; **P ≤ 0.01; ***P ≤ 0.001). [file mmc6.pdf]

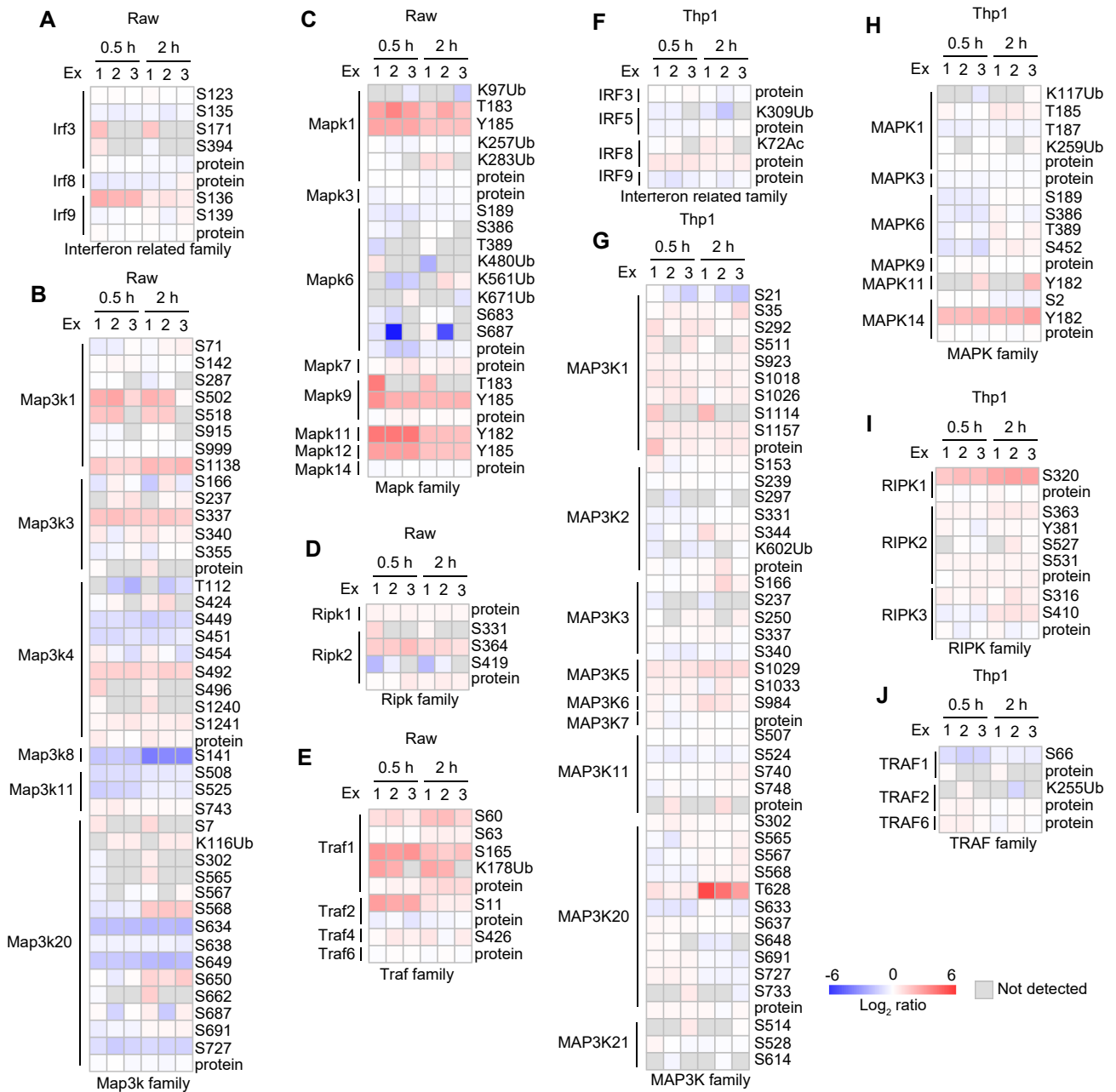

Supplement: Supplementary Figure S7 — Regulated proteins involved in inflammatory signaling pathways after cells were stimulated with LPS The diagram shows the intensity of signals for proteins and PTM sites involved in IRF (A, F), MAP3K (B, G), MAPK (C, H), RIPK (D, I), and TRAF (E, J) inflammatory signaling pathways identified in cells stimulated with LPS for 0.5 hours and 2 hours. [file mmc7.pdf]
