# Supplementary material for: Integrative Proteomic Analysis of Multiple Posttranslational Modifications in Inflammatory Response
Source: Genomics Proteomics Bioinformatics. 2021 Mar 2;20(1):163–76. doi: 10.1016/j.gpb.2020.11.004 (PMC9510875; doi:10.1016/j.gpb.2020.11.004)
Supplement: Supplementary Figure S8 — A database of PTM in inflammation Map2k3 served as an example of the PTM-inflammation website (http://ptm-inflammation.cn). The figure shows basic information about the protein, such as the sub-cellular location, modification type, UniProt description, sequence, etc. It also includes a visualization of post-translational data and a table providing detailed data. [file mmc8.pdf]

Basic information for Map2k3

Uniprot ID	O09110
Symbol Name	Map2k3
Organism	mouse
Mass (Da)	39296
Length (aa)	347
Subcellular Location	unknow
Modification Type	unknow
Uniprot Description	Dual specificity kinase. Is activated by cytokines and environmental stress in vivo. Catalyzes the concomitant phosphorylation of a threonine and a tyrosine residue in the MAP kinase p38. Par... Show more
Sequence	MESPAASPPASLPQTKGKSKRKKDLRISCVSKPPVSNPTPPRNLDSRTFITIGDRNFEVEADDLVTISELGRG... Show more

Plot for Map2k3

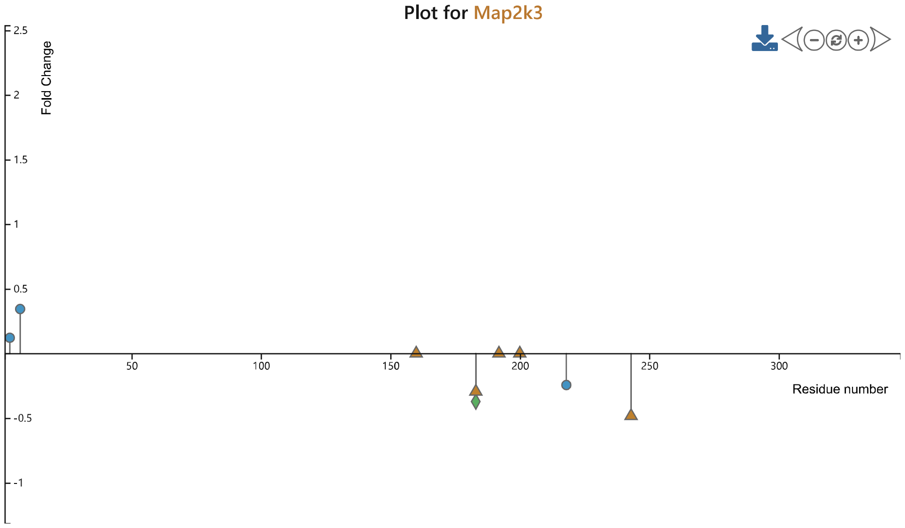

Conditions

- 30min
- 120min
- 30min+mg132
- 120min+mg132

PTM sites

- Phosphorylation
- Acetylation
- Ubiquitylation

Detail table

Position	Type	Flanking Sequen...	30min	120min	30min+mg132	120min+mg...
S3	PHOSPHORYLAT...	______MEsPAAS...	0.121	0.015	0.000	0.000
S7	PHOSPHORYLAT...	_MESPAAsPPAS...	0.343	2.116	0.000	0.000
K160	UBIQUITYLATI...	KVLEKNMkIPED...	0.000	0.000	-0.370	0.182
K183	ACETYLATION	ALEHLHSkLSVI...	-0.374	-0.732	0.000	0.000
K183	UBIQUITYLATI...	ALEHLHSkLSVI...	-0.295	0.117	-0.413	-0.079
K192	UBIQUITYLATI...	SVIHRDVkPSNV...	0.000	0.000	-1.094	-0.233
K200	UBIQUITYLATI...	PSNVLINkEGHV...	0.000	0.000	-0.793	0.100
S218	PHOSPHORYLAT...	ISGYLVDsVAKT...	-0.245	-0.004	0.000	0.000
K243	UBIQUITYLATI...	INPELNQkGYNV...	-0.484	0.043	-0.595	-0.013

Previous Page 1 of 1 10 rows Next
